# Lack of insurance coverage and urgent care use for asthma: A retrospective cohort study

**DOI:** 10.1186/1471-2458-6-14

**Published:** 2006-01-24

**Authors:** Barry P Markovitz, Elena M Andresen

**Affiliations:** 1 Washington University School of Medicine, St. Louis, MO, USA; 2 Department of Veterans Affairs Medical Center/University of Florida College of Public Health and Health Professions, Gainesville, FL, USA

## Abstract

**Background:**

Asthma is a common chronic disease with profound impacts upon individuals and the US health care system. Inadequate health care coverage has been associated with more frequent and severe exacerbations of the disease. We examined the relationship between adequacy of health care coverage and use of emergent care of adults with asthma.

**Methods:**

The 2001 Behavioral Risk Factor Surveillance System was the source of data on adults with current asthma. Bivariate and multiple logistic regression analysis modeled identifiable factors in predicting urgent or emergent care.

**Results:**

Key variables included demographics and information on self-reported gaps in health care coverage. The primary outcome was emergency room or urgent care visits for worsening of asthma symptoms. Of 16,234 subjects nationally with current asthma, 2,195 from eight states had valid responses to a supplemental module asking about emergency room use or urgent care visits because of asthma. Thirty four percent of these individuals required such care in the previous year. Having an interruption in health care coverage in the past year was associated with an increased risk of needed urgent or emergent care (crude Odds Ratio [OR] 1.48, 95% confidence intervals [CI]1.03, 2.1). The association was not statistically significant in the adjusted multivariate model including race/ethnicity, employment status, gender, age, education and the ability to identify a primary physician (adjusted OR 1.2, 95% CI 0.8, 1.8).

**Conclusion:**

This study provides population-level, generalizable evidence of increased risk of exacerbations of asthma in adults and (1) their demographic characteristics, and (2) continuous adequate health care coverage.

## Background

Asthma is a chronic illness affecting an estimated 7.2% of adults in the U.S. with significant morbidity and mortality [1]. An estimated 4,487 deaths, 1.8 million emergency department visits, and 10.4 million physician office visits were attributed to asthma in 2000. Asthma is one of a number of chronic conditions that, improperly managed by regular outpatient care, results in increased morbidity, use of emergency services and inpatient hospitalization [2]. Adequate insurance coverage in patients with asthma correlates with reduced use of urgent or emergent care. For example, Davidson found children on Medicaid far more likely to utilize emergency room care, compared to children with adequate private insurance [3]. Impaired access to health care – strongly correlated with socioeconomic status – has been and continues to be a major impediment to optimal care for many individuals with such chronic diseases and consequently is likely to be related to the increased use of emergency care [4]. In his paper, Andrulis summarizes a large body of evidence to this effect and the corollary that reducing health care access disparities improves outcomes of those with chronic diseases such as asthma.

The Behavioral Risk Factor Surveillance Survey (BRFSS) is a state-based, telephone (random-digit-dialed) survey of the noninstitutionalized U.S. population aged 18 years of age and older, on data related to chronic diseases and their risk factors [5,6]. This survey provides annual population-based cross sectional data that can be used to track self-reported risks and health conditions over time and allow for testing of hypotheses regarding such risks and disease outcomes. The BRFSS includes national universal questions and modules, and, as in 2001, state-added modules on special topics of interest to these states. We used the BRFSS 2001 to assess the relationship between access to health care and short-term asthma outcomes. Specifically we asked whether any interruption in health care coverage resulted in the use of more urgent or emergency care for subjects with asthma.

## Methods

The population studied included those respondents to the BRFSS 2001 with affirmative answers to the two asthma questions (asked in all 50 states, the District of Columbia and U.S. Territories): "Have you ever been told by a doctor, nurse, or other health professional that you have asthma?" and "Do you still have asthma?" An additional series of questions regarding asthma was asked of participants as a state-added module in eight states in 2001; to be included in this analysis, a valid response to one or both of the following questions was required: "During the past 12 months, how many times did you visit an emergency room (ER) or urgent care center because of your asthma?" and "During the past 12 months, how many times did you see a doctor, nurse, or other health professional for urgent treatment of worsening asthma symptoms?" Responses to these questions were combined and the dichotomous variable created as a yes or no reply to either or both of these original questions regarding urgent or emergency care for asthma. The eight states who chose to use the asthma care module represented a broad range of the U.S. population (Indiana, Iowa, Michigan, Mississippi, Missouri, Pennsylvania, South Dakota, Washington).

The primary predictor variable was the reply to the question: "During the past 12 months, was there any time that you did not have any health insurance or coverage?" The additional potential confounding variables analyzed included age (recorded as 18–24, then 5 year epochs, then ≥ 80 years of age; the 13 categories were then analyzed as a continuous variable; age also was analyzed as 18–64 vs. ≥ 65 years), gender, race/ethnicity (white non-Hispanic, black non-Hispanic, others non-Hispanic, Hispanic), employment status (employed, homemaker/ student/retired, unemployed/can't work), education (< high school graduate, high school graduate, college graduate), income (< $10,000/year, $10–50,000/year and > $50,000/year), marital status (married vs. not married) and response to the questions "Do you have one person you think of as your personal doctor or health care provider?" and "Was there a time during the last 12 months when you needed to see a doctor, but could not because of the cost?"

Descriptive analyses compared characteristics of those who had or did not have urgent care/ER use. Bivariate analysis of categorical variables with outcome was tested with chi-square and ranked ordinal variables by chi-square for trend (p < 0.05 considered significant); unadjusted odds ratios (OR) with 95% confidence intervals (CI) are presented. Logistic regression was performed with odds ratios and 95% confidence intervals reported. Logistic regression used "coverage" as the primary exposure, and included any additional variable if it had a meaningful effect on the odds ratio of coverage (10% or more change in the OR), or if the additional variable itself was a significant predictor of urgent care/ER use. Effect modification by age (dichotomized at 65) was also examined using a liberal p-value for interaction of 0.10. All analyses were performed with SPSS 11.0 for Macintosh for unweighted data (SPSS, Inc., Chicago, IL, 2002). The study was approved by the St. Louis University Institutional Review Board.

## Results

There were 24,067 affirmative replies (11.33% of total respondents) to the first asthma question ("..ever been told you have asthma") and 16,324 reported still having asthma. Of these subjects, 2,195 individuals had valid responses to the questions regarding urgent or emergency room use in the past year; of this group, 34% of subjects needed such care in the previous year. Comparison of the subjects from the BRFSS focused sample of eight states showed no differences health coverage, health behaviors, and demographic factors except for a slightly higher percentage of respondents who were white/non-Hispanic in the smaller group (83% in the eight-state sample versus 76% in the BRFSS national sample of persons with asthma: chi square = 59.8, p < 0.001). Table 1 illustrates bivariate analysis of the relationship between individual predictors and the use of urgent or emergency room care. A positive reply to interruption in health care coverage was associated with an increased risk of needing urgent or emergency room care (OR 1.48, 95% CI 1.03, 2.1), as was black/non-Hispanic race (OR 2.24, 95% CI 1.63, 3.0), other non-Hispanic race (OR 1.81, 95% CI 1.24, 2.6), and age (18–64, OR 1.77, 95% CI 1.37, 2.28), and a positive response to the inability to see a physician in the past year due to cost (1.19, 95% CI 1.08, 2.95). In addition, male gender was associated with a reduced risk of needing urgent or emergent care (OR 0.59, 95% CI 0.48, 0.72) and being unemployed/can't work carried an increased risk (OR 1.56, 95% CI 1.24, 1.97).

A series of logistic regression models were evaluated to examine potentially confounding factors and the most parsimonious model is presented in Table 2. Once race/ethnicity was entered into the model, the odds ratio of the primary predictor of interest was not statistically significant at 1.2 (95% CI 0.8, 1.8) and varied little irrespective of adding or subtracting additional variables.

Retaining a significant effect were black/non-Hispanic race (OR 2.4, 95% CI 1.7, 3.4), other/non-Hispanic race (OR 2.1, 95% CI 1.4, 3.1), male gender (OR 0.6, 95% CI 0.5,0.7), and unemployed/can't work status (OR 1.6, 95% CI 1.2, 2.1). Although subjects above the age of 65 required more urgent or emergent care for asthma in the bivariate analysis, age, whether entered as a ranked variable (in 5 year increments) or as dichotomous (older or younger than age 65), was not a significant predictor of urgent or emergent care use in multivariable analysis.

## Discussion

In our descriptive analysis, a break in health care coverage in the past year was associated with the use of urgent or emergent care for asthma. In addition to the burden of asthma on society, there is substantial impairment of the quality of life in individuals with current disease, as shown in work by Ford et al. using the BRFSS 2000 survey [7]. They found that adults with asthma experienced twice the number of impaired physical or mental health days each month compared to those without asthma. More than one third of the subjects in the sample in our report required urgent or emergency room care at least once in the past year for their disease.

Lack of or interruptions of health care coverage has been demonstrated to be associated with increased use of emergency care, as emergency rooms become the health care provider of last resort for those without insurance, and therefore without proper maintenance or preventative care [4]. As Andrulis discusses, socioeconomic status and race are also well-established markers of poorer health care outcomes, but there is evidence that once access to health care is leveled across class and race, these differences are markedly reduced. He cites several studies that support this paradigm: having adequate insurance ensures access to regular care which reduces the use of emergency care, as a large portion of emergency room visits are for non-urgent care by individuals without adequate insurance. Since passage of the Emergency Medical Treatment and Active Labor Act (EMTALA) in 1985, no one can be denied emergency room care in the U.S. for lack of ability to pay. Additional support is evident in survey of almost 500 nonelderly adults with asthma in California, where a correlation was demonstrated between insurance status (Medi-Cal), access to regular care, and the use of emergency room visits for asthma symptoms [8].

However, not all the evidence is consistent as efforts are made to extend health care coverage to more groups with government programs. New York was an early developer of the Child Health Insurance Program (CHIP) that provided coverage to children ineligible for existing Medicaid and without other insurance. Szilagyi et al. found substantial improvements in the health care of children with asthma associated with this program [9]. Finkelstein compared 1,928 Medicaid-covered with 11,007 privately-insured children with asthma within the same HMO and found Medicaid-insured children 1.4 times as likely to receive emergency room care (95% CI 1.2, 1.5) and 1.3 times as likely to be hospitalized (95% CI 1.1, 1.5) as non-Medicaid-insured within this HMO [10].

In our descriptive univariate analysis, a break in health care coverage was a significant, albeit modest, predictor of needing urgent or emergency room care in the previous year. This relationship became non-significant once race/ethnicity was considered in the regression model. In an ad hoc analysis, we considered whether minority status might operate as an effect modifier (interaction) of health insurance gap. While the health gap effect in minorities was greater than for whites, the difference was not significant. This analysis could not confirm that the effect was statistically significantly different by (modified by) race/ethnicity. This potential should be examined in future studies, since it is plausible that the effect of inconsistent coverage has a larger effect for minority adults in the U.S. In our analysis, Blacks and other minorities were more likely to have a gap in insurance coverage (data not shown) and were also more likely to require urgent or emergent care; race/ethnicity is, therefore, a classic confounder in this analysis. Race/ethnicity is likely to be a marker for a constellation of factors rather than a risk factor in and of itself. Race/ethnicity is also potentially associated with more severe disease, although we could not determine that from the BRFSS. Thus, it is an important adjustment factor in order to observe the independent effect of a gap in coverage. Interestingly, the role of having a single physician identified as the primary health care provider did not appear to play a role in this analysis. It may be that using a single emergency room or urgent care visit as the primary outcome measure is too insensitive a measure to distinguish the role of a gap in health care coverage. However, a separate model using just emergency room care as the dependent variable showed little differences in the predictors' effects (results not shown).

One of the limitations of this study is the reduced power with only 2,195 subjects with complete data for analysis, and only 131 subjects had a gap in health insurance coverage in the previous year. With a modest effect size of OR = 1.2, this study had less than 10% power to detect a significant effect. A larger study with over 5,000 respondents (with a health insurance gap) would be required to confirm the relationship reported here. Further, because BRFSS does not sample persons aged <18 years or persons who are in institutions, who are in households without a telephone, and potentially under samples those who are hearing impaired, who have cognitive, speech, and other communication impairments, or who have limited stamina and cannot get to the telephone, findings in this report cannot be generalized to the individual or aggregate state populations without some limitation. In addition, only 8 states are included in this report. While the subset of people with asthma described here vary minimally from the national BRFSS, some caution should be used in generalizing to other states. The considerable advantage of data from the BRFSS is the random selection of community-dwelling adults, which allow for population inferences that are not present in analyses of health plan enrollees and clinical databases. In addition, the BRFSS provides self-reported data that are not limited to process, billing, encounter, or utilization variables recorded in electronic databases.

We did not have detailed information about the underlying medical severity of asthma of the subjects. In addition, the response rate to the BRFSS overall in 2001 was only 51%, and there may be systematic differences between those who replied to the survey in these matters and those who did not [1]. The BRFSS survey data are examined for quality, and some biases are noted in demographics of respondents [11]. For our analyses, if those who did not respond were more likely to need urgent care and to have inadequate heath care coverage, our estimate is biased toward the null, or no effect. However, even a moderate relative relationship would translate to substantial public health impact because of the large number of people affected.

## Conclusion

In this study, 7% of respondents with asthma had a break in coverage, and among these, over 43% required urgent or emergency room care compared to 34% of all subjects with asthma. With an estimated 14.7 million adults with asthma in the U.S., cautious extrapolation suggests that upwards of 80,000 individuals may experience at least one additional urgent care visit associated with inadequate health care coverage. Finally, these results suggest that racial/ethnic differences in the use of urgent care for asthma are not related only to problems in health care coverage. These disparities need to be examined in more detail in future research – where the effects of race/ethnicity and severity of illness can be measured more precisely, and their effects isolated better – on access to care and asthma care.

## Competing interests

The author(s) declare that they have no competing interests.

## Authors' contributions

BPM and EMA both participated in the design, data analysis and manuscript preparation of this study.

## Pre-publication history

The pre-publication history for this paper can be accessed here:



## Figures and Tables

**Table 1:** Use of urgent or emergency asthma care in the 2001 Behavioral Risk Factor Surveillance System (8 states; n = 2,195 with valid replies)

		Required urgent or emergency room care for asthma in past 12 months		crude OR (95% CI)	Total
		
		Yes (%)	No (%)		
Health care coverage gap in past year? *	Yes	56 (8)	75 (6)	1.48 (1.03,2.1)	131
	No	611 (92)	1210 (94)		1821
	Totals	667 (100)	1285 (100)		1952
Gender *	Men	172 (23)	482 (33)	0.59 (0.48, 0.72)	654
	Women	580 (77)	961 (67)		1541
	Totals	752 (100)	1443 (100)		2195
One physician identified as primary?	Yes	606 (81)	1120 (78)	1.20 (1.0,1.5)	1726
	No	146 (19)	320 (22)		466
	Totals	752 (100)	1440 (100)		2192
Health plan in past year	Yes	667 (89)	1288 (89)	0.95 (0.71,1.26)	1955
	No	83 (11)	152 (11)		235
	Totals	750 (100)	1440 (100)		2190
Race/ethnicity *	White/non-Hispanic	576 (77)	1235 (87)	reference	1811
	Black/non-Hispanic	92 (12)	88 (6)	2.24 (1.63,3.0)	180
	Other/non-Hispanic	59 (8)	70 (5)	1.81 (1.24,2.6)	129
	Hispanic**	23 (3)	34 (2)	1.45 (0.83,2.5)	57
	Totals	750 (100)	1427 (100)		2177
Age group*	18 to 64	658 (88)	1147 (80)	1.77 (1.37,2.28)	1805
	65 and older	92 (12)	284 (20)		376
	Totals	750 (100)	1431 (100)		2181
Too costly to see physician in past year*	Yes	31 (19)	43 (11)	1.79 (1.08,2.95)	74
	No	136 (81)	337 (98)		473
	Totals	167 (100)	380 (100)		547
Income *	< $10,000	83 (12)	118 (9)	reference	201
	$10–50,000	464 (67)	801 (64)	0.82 (0.60,1.13)	1265
	> $50,000	150 (22)	331 (27)	0.64 (0.45, 0.92)	481
	Totals	697 (100)	1250 (100)		1947
Employment *	Employed	411 (55)	816 (57)	reference	1227
	Homemaker/student/retired	169 (23)	411 (29)	0.81 (0.66, 1.01)	580
	Unemployed/can't work	172 (23)	214(15)	1.56 (1.24,1.97)	386
	Totals	752 (100)	1441 (100)		2193
Education	< HS graduate	108 (14)	198 (14)	reference	306
	HS graduate	462 (61)	902 (63)	0.94 (0.72,1.22)	1364
	College graduate	182(24)	338 (24)	1.01 (0.75, 1.36)	520
	Totals	752 (100)	1438 (100)		2190
Marital status	Married	345 (46)	700 (49)	0.90 (0.75,1.07)	1045
	Not married	406 (54)	739 (51)		1145
	Totals	751 (100)	1439 (100)		2190
Smoke now?	Yes	233 (55)	394 (50)	1.22 (0.97,1.55)	627
	No	194 (45)	401 (50)		595
	Totals	427 (100)	795 (100)		1222

* p < 0.05 by chi-square analysis

** small sample size may limit interpretation of results for Hispanic subjects

HS = High school, OR = odds ratio, CI = confidence interval

**Table 2:** Logistic regression model of predictors of needing urgent or emergency room care in past year in subjects with asthma from the 2001 Behavioral Risk Factor Surveillance System (8 states; n = 2,195)

		Adjusted OR*	95% CI
		
Race/ethnicity	White/non-Hispanic	reference	---
	Black/non-Hispanic	2.4	1.7,3.4
	Other/non-Hispanic	2.1	1.4,3.1
	Hispanic	1.1	0.6,2.0
Health care coverage gap in past year?	No	reference	---
	Yes	1.2	0.8,1.8
Gender	Female	reference	---
	Male	0.6	0.5,0.7
Age	(per 5 year increase)	1.0	0.9,1.0
Education	College graduate	reference	---
	< High school graduate	0.9	0.6,1.2
	High school graduate	0.9	0.7,1.1
Employment status	Employed	reference	---
	Homemaker/student/retired	0.9	0.7,1.1
	Unemployed/can't work	1.6	1.2,2.1
One physician identified?	No	Reference	---
	Yes	1.2	1.0,1.6

* Odds ratios adjusted for all other variables in the model

CI = confidence interval

## References

[B1] Centers for Disease Control and Prevention (2003). Self-reported asthma prevalence and control among adults – United States, 2001. JAMA.

[B2] Strunk RC, Ford JG, Taggart V (2002). Reducing disparities in asthma care: priorities for research – National Heart, Lung, and Blood Institute workshop report. J Allergy Clin Immunol.

[B3] Davidson AE, Klein DE, Settipane GA, Alario AJ (1994). Access to care among children visiting the emergency room with acute exacerbations of asthma. Ann Allergy.

[B4] Andrulis DP (1998). Access to care is the centerpiece in the elimination of socioeconomicdisparities in health. Ann Int Med.

[B5] Gentry EM, Kalsbeek WD, Hogelin GC, Jones JT, Gaines KL, Forman MR, Marks JS, Trowbridge FL (1985). The behavioral risk factor surveys: II. Design, methods, and estimates fromcombined state data. Am J Prev Med.

[B6] Remington PL, Smith MY, Williamson DF, Anda RF, Gentry EM, Hogelin GC (1988). Design, characteristics, and usefulness of state-based behavioral risk factor surveillance: 1981–87. Public Health Rep.

[B7] Ford ES, Mannino DM, Homa DM, Gwynn C, Redd SC, Moriarty DG, Mokdad AH (2003). Self-reported asthma and health-related quality of life: findings from the behavioral risk factorsurveillance system. Chest.

[B8] Bair YA, Garcia JA, Romano PS, Siefkin AD, Kravitz RL (2001). Does "mainstreaming" guarantee access to care for medicaid recipients with asthma?. J Gen Intern Med.

[B9] Szilagyi PG, Holl JL, Rodewald LE, Yoos L, Zwanziger J, Shone LP, Mukamel DB, Trafton S, Dick AW, Raubertas RF (2000). Evaluation of New York State's Child Health Plus: childrenwho have asthma. Pediatrics.

[B10] Finkelstein JA, Barton MB, Donahue JG, Algatt-Bergstrom P, Markson LE, Platt R (2000). Comparing asthma care for Medicaid and non-Medicaid children in a health maintenance organization. Arch Pediatr Adolesc Med.

[B11] Centers for Disease Control and Prevention http://www.cdc.gov/brfss/pdf/99quality.pdf.

